# Integrating Cyber-Physical Systems in a Component-Based Approach for Smart Homes

**DOI:** 10.3390/s18072156

**Published:** 2018-07-04

**Authors:** Javier Criado, José Andrés Asensio, Nicolás Padilla, Luis Iribarne

**Affiliations:** Applied Computing Group, University of Almería, 04120 La Cañada de San Urbano, Spain; jacortes@ual.es (J.A.A.); npadilla@ual.es (N.P.); luis.iribarne@ual.es (L.I.)

**Keywords:** smart homes, cyber-physical systems, sensors, interoperability, component-based applications, COScore

## Abstract

Integration of different cyber-physical systems involves a development process that takes into account some solutions for intercommunicating and interoperating heterogeneous devices. Each device can be managed as a thing within the Internet-of-Things concept by using web technologies. In addition, a “thing” can be managed as an encapsulated component by applying component-based software engineering principles. Based on this context, we propose a solution for integrating heterogeneous systems using a specific component-based technology. Specifically, we focus on enabling the connection of different types of subsystems present in smart home solutions. This technology enables interoperability by applying a homogeneous component representation that provides communication features through web sockets, and by implementing gateways in proprietary network connections. Furthermore, our solution eases the extension of these systems by means of abstract representations of the architectures and devices that form part of them. The approach is validated through an example scenario with different subsystems of a smart home solution.

## 1. Introduction

With the increasing development of technological devices, more and more communication capabilities are being provided. Most devices (from switches, LEDs and air conditioners to televisions, speakers and watches) are now or will be soon connected to the Internet [1] and some research works estimated that the Internet of Things will have about 24 billion connected devices by 2020 [2], not only through mature wireless technologies such as bluetooth, ZigBee, Z-Wave or WiFi, but also due to new emerging communication standards [3]. The concept of Internet-of-Things (IoT) can be analyzed within the bigger domain of Cyber-Physical Systems (CPS), since IoT approaches represent subsets of this domain, including the connectivity between devices, the application of smart grids, or the availability of sensor information through the Web, among other possible examples [4]. Nevertheless, the IoT concept is more related to opening and connecting smart devices, whereas the CPS term is related to the physical processes, applicability and problem solving of complete systems [1].

In this kind of system, sensors are the main source of information and, therefore, the obtained data should be shared with the rest of the parts that require it [5]. In addition, not only information captured by physical devices must be taken into account, but also data received or gathered by software components should be considered [6,7]. This type of virtual sensor can supplement the behavior of existing physical devices or emulate ones that are not present, with the aim of evaluating the tasks performed by existing or future installations [8,9]. In this sense, interoperability is a key problem concerned with those specific solutions having the need of maximizing the performance of communications among sensors and the rest of devices of an installation and/or between devices belonging to different systems [10]. This is even more relevant in the case of heterogeneous systems with different communication protocols, which may imply the use of a middleware [11].

Regardless of the domain, we can find examples of interoperability solutions based on common operating systems (such as Contiki, RIOS, FreeRTOS or TinyOS), supported by a middleware in terms of a programming language [12], offering a high-level Application Programming Interface (API) to transparently access the heterogeneous devices [13], or solved by multi-agent system (MAS) middlewares [14]. With respect to the life-cycle, approaches and mechanisms supported by Component-Based Software Engineering (CBSE) are useful to design, develop and maintain IoT and cyber-physical systems, and these techniques can be applied to ease interoperability [15]. The reason is not only related to the abstract definition of physical components and their connections but also because of the positive impact of software components to resolve the heterogeneity in modeling, communicating and extending tasks. In this sense, some technical barriers such as the heterogeneity of models, development tools and life-cycle management can be addressed with CBSE technologies [16].

**Figure 1:**
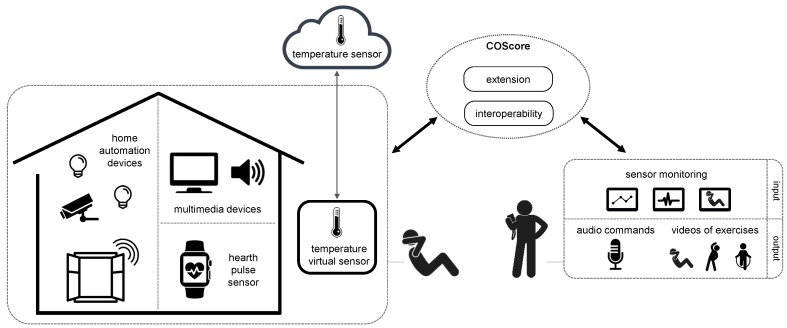
Heterogeneous cyber-physical systems in an example scenario.

We can see this heterogeneity in an example scenario of remotely monitoring the fitness activity of a person who is in his/her house (see Figure 1). This character (left side of the figure) is living in a smart home with different cyber-physical systems. First, a number of light-bulbs, a security camera and a sensor to detect the opening of a window are connected in a home automation system. Second, a smart TV and a set of wireless speakers form part of a multimedia system. Third, a smart watch is another CPS intended to obtain the heart rate of the character and, finally, a virtual sensor is reading the outside temperature, which is acquired from a third-party service available in the cloud.

All the mentioned systems use different technologies and communication protocols and they cannot be easily connected to perform a common task. In this case, the goal of the scenario is to connect the customer (the character at home) with a personal trainer (right side of Figure 1). The trainer will receive the temperature data, heart rate and images from the camera as useful information to monitor the activity. This character can send audio commands that will be played through the speakers and they are also able to send videos of new exercises that will be shown in the smart TV. Furthermore, this behavior must coexist with the security task of the home automation system that will sound an alarm signal through the speakers and will blink the light-bulbs if the window sensor is activated.

The main problem here is to allow the connection between the different parts, thus converting each system in a subsystem of a whole solution which enables the interoperability. In addition to the problem of developing a solution which interconnects all the subsystems, the entire system of the example scenario cannot be easily extended if a new device must be incorporated or if we want to improve the involved tasks of the system. For example, if we want to add a new behavior which changes the focus of the security camera from the monitored character to the window in order to visualize the source of the sensor activation, we must re-implement the behavior of the camera and TV controllers and, consequently, evaluate that related systems are working properly. Depending on the technologies used in each system, this extension could become a process with high costs derived from new design and construction activities.

This article proposes an approach based on CBSE and modeling techniques to develop solutions of CPSs in general and smart homes in particular. The proposal is focused on enabling the interoperability between heterogeneous devices belonging to the same system and also between heterogeneous CPSs. In particular, our approach includes design and implementation principles based on a component technology called COScore [17], which assists the extension of this kind of systems by modifying their underlying software architecture through model abstractions. Therefore, our approach is intended to facilitate the mentioned development tasks but also the execution of a system behavior acting as an intermediary between the systems (see Figure 1). Furthermore, this middleware proposes two functioning modes: (1) a global mode when the connection to the Internet is available and distributed systems can be interrelated; and (2) a local mode when a system is isolated (maybe because the Internet connection fails) and our solution must provide a normal execution inside the local network. In summary, the main contributions of the paper are:

- Design principles to implement different solutions for interoperability of physical and virtual sensors with the rest of devices of an installation have been proposed.
- A development based on software components is applied to encapsulate the structure and behavior of devices, thus organizing the implementation and enabling the reuse.
- The representation of these components in terms of models helps to formalize the definitions and allows for generating (completely or partially) the smart home applications.
- A back-end infrastructure offering the available operations as web services supports the management of the architectures and components.
- The communication between devices is accomplished through an homogeneous layer by using web technologies.
- Modularity properties (coupling and cohesion) have been analyzed to determine the division of a smart home solution into different subsystems.
- Architecture and component models for defining this kind of the solution are described by an example scenario.
- Different alternatives of communication in these solutions are identified and exemplified with the corresponding data flows.
- Implementation examples of gateways which enable the communication with proprietary technologies are described.

The rest of the paper is organized as follows. Section 2 describes the required background information to understand our component-based technology. Section 3 proposes a software solution to provide interoperability in CPSs described under the principles of our kind of software architectures. Section 4 illustrates the applicability of this solution by means of an example scenario, which includes descriptions of different kinds of components that are present in a CPS, and an interaction example of monitoring the fitness activity of a user. Section 5 reviews the most relevant related work. Finally, conclusions and future work are drawn in Section 6. This section also discusses the contributions and the threats to the validity of our approach.

## 2. Background of Our Technology

As discussed in the previous section, to support the extension and inclusion of different types of CPS, we have developed our own technological infrastructure (called COScore). Its development is based on CBSE, Model-Driven Engineering (MDE), and Cloud Computing-based technology, and it is widely described in [17]. In order to facilitate the understanding of this article, it is necessary to summarize the most relevant aspects of this infrastructure, which are described below.

**Figure 2:**
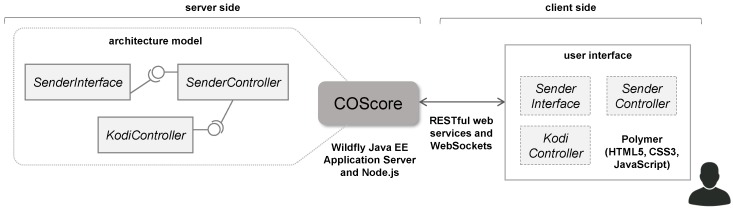
Main parts of the background technology (COScore).

Let us consider the Figure 2. Our proposal requires that each user application must be defined as a set of independent components, available in a variety of repositories (ownership or third-party). We have called these components as COTSgets, from Commercial Off-The-Shelf (COTS) [18] and gadGETS. Following the previous example, let’s suppose that a personal trainer wants to send a resource (audio, video, etc.) to a customer for playing it with a KODI multimedia center. KODI is an open source software that provides a friendly user interface to play (in addition to record, schedule, serve, etc.) multimedia files stored in a local storage or available from a remote web URL. Moreover, all the actions can be executed through an API instead of interacting with the user interface (https://kodi.tv/). In this case, we can create three components: *KodiController*, *SenderController*, and *SenderInterface* (right side of Figure 2). The first component is used for the interaction with KODI; therefore, it is responsible for managing the communication. The other two components are responsible for requesting the trainer the resource to be sent to KODI. One of them is responsible for managing the user interface (*SenderInterface*), and the other (*SenderController*) is responsible for sending the information to *KodiController* so that the URL of the resource can be transmitted. This division of these last two components allows us to build different interface components (e.g., for different devices) and have a single controller component. Regarding the technological aspects, the user application components are implemented as web components using the Polymer technology [19], which offers the following advantages: encapsulation of components implementation, HTML templates to develop components, reuse of components by initializing with different parameters, and the use of well-known web technologies (HTML5, CSS3 and JavaScripts files) for the implementation. A component example implemented with Polymer is available in https://github.com/acgtic211/nha_video_viewer_interface.

All user application components are grouped forming an architecture (left side of Figure 2). COScore is used for the deployment of these architectures offering several operations, including the management of the COTSgets specifications, the management of the COTSgets-based architectures, the instantiation of COTSgets components, the initialization of user applications based on the architectures, and the communication of components belonging to an architecture. All these capabilities are offered at run-time through web services. Regarding the technological aspects, COScore has been developed as a cloud service. It includes a JavaScript server (implemented with Node.js) used as a link with the user applications, and it makes use of WebSockets [20] (which requires the Socket.IO library [21]) to manage the communications. This way, the components forming the architectures can be communicated with each other by exchanging messages by using web sockets. Also, a Wildfly application server has been deployed (http://wildfly.org) in COScore for providing the services that are valid for all the platforms. Its functionalities are based on the component descriptions and their relationships, regardless of the platform on which they are deployed. This application server offers a set of RESTful web services developed with JAX-RS, which are called up by HTTP requests.

**Figure 3:**
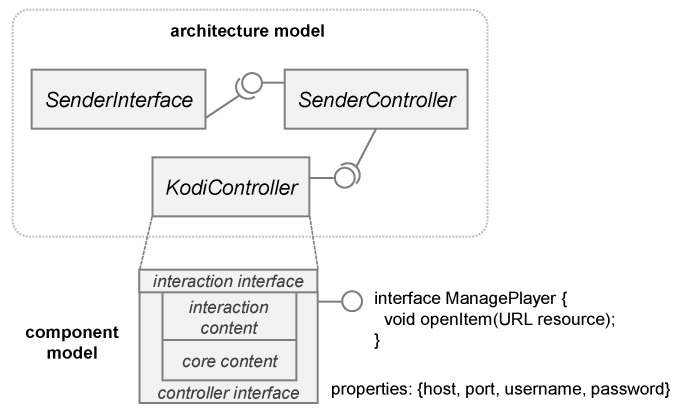
Component model required by the COScore technology.

Each component is structured internally in four parts to define its functionality and interfaces (see Figure 3): (1) the *interaction content*; (2) the *core content*; (3) the *interaction interface*; and (4) the *controller interface*. Regarding the functionality, a component includes the *interaction content* that is used for implementing the business logic of the interaction with the user, and the *core content* (which is the main part of a component) to store the methods implementing the rest of a component behavior. The two interfaces are the *interaction interface* storing the methods for handling interaction events with the user, and the *controller interface* that is used for managing the communication with other components. In our approach, the operations of a component are accessible from the outside through both the interaction and controller interfaces.

All the components need to use the controller interface, but depending on the rest of implemented parts, we can pick out different types of components: (a) the *container component*, which identifies a component that contains other components, making it possible to build complex components from more basic ones; (b) the *functional component* (with *core content*), which is used to construct functional components without user interaction, and therefore, can be built to execute background code; (c) the *user interaction component*, which is used to build components that include user interaction (using *interaction interface* and *interaction content*) or simply display information (with *interaction interface*); and finally, (d) the *normal component* (with all the parts), which is the union of functional and *user interaction* component types, and therefore, it is a component that includes interaction with the user and the internal functionality of the component.

Also, each component contains a list of properties. Properties may provide information on non-functional properties (NFPs) (i.e., quality of service (QoS), component appearance, such as width, height, etc.) and any dependencies on other components. Some of these properties can be modified and should be taken into account at run-time. For example, four properties defined by the component *KodiController* are: (1) *host*, used to identify the host where KODI is installed; (2) *port*, used to indicate the port for the remote control of KODI via HTTP; (3) *username* and (4) *password*, used to indicate the user name and password to get control of KODI.

As indicated above, the controller interface is used for managing the communication with other components. It is comprised of a set of provided and required interfaces, with the condition that each component must have at least one provided interface. The provided interfaces define all the component functionalities visible to the outside world, i.e., it describes methods that can be invoked to initiate the execution of some operation. The required interfaces describe the operations belonging to other components invoked by a component for its proper operation. In the example, the component *SenderInterface* defines the required interface *ManageItem* to send a resource selected by the user to KODI. In order to receive the resource, the component *SenderController* defines the provided interface *ManageItem*. Next, the required interface *ManageItem* belonging to this component will send the resource to the provided interface *ManagePlayer* belonging to the component *KodiController*. With this summary of the most relevant aspects of our infrastructure, we can now describe how we achieve interoperability between different CPSs.

## 3. Interoperability in Cyber-Physical Systems

**Figure 4:**
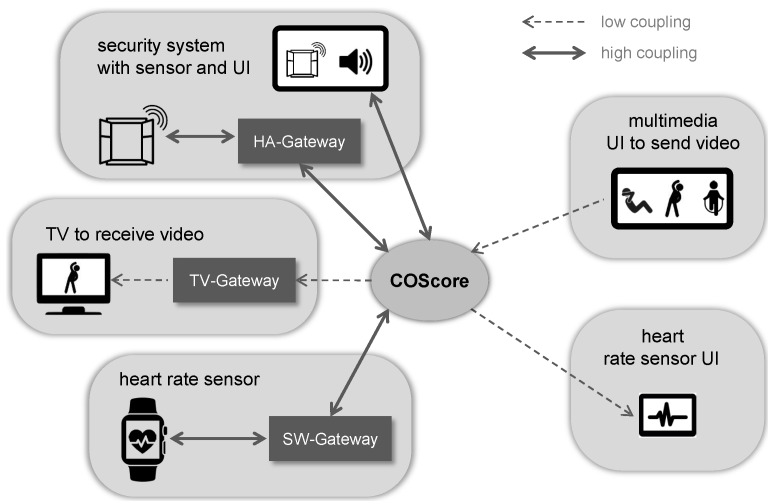
Connection of cyber-physical systems in a smart home solution.

The communication between heterogeneous CPSs requires the use of mechanisms supporting the connection of the different protocols and technologies related to the devices forming each system. With this aim, our component-based technology provides the models and abstract definitions needed to describe the architectural structure and the involved components. In addition to these models, it is necessary to formally define a set of principles and rules to enable the interoperability in CPSs thus driving the development and maintenance of such systems. To describe these principles, this section comes back to the previous example scenario of Figure 1 but focused on an excerpt. The personal trainer interacts with two systems: a user interface (UI) showing the heart rate information obtained from the monitored user, and a UI to send new videos with exercises. Other three systems are present in the side of the monitored customer. First, a smart watch is sending data from a heart rate sensor and it also receives information to show it in the device. Second, a television plays the videos received by the trainer. Third, a security system of home automation devices is in charge of controlling if the window has detected any intruder and warning the user through a graphical interface (see Figure 4).

### Different Solutions for the Interoperability

As shown in Figure 4, different types of connection can be established to solve the interoperability in CPS. Depending on the number of interconnections between a system and the COScore, we have defined two types of relations: low coupling and high coupling. The first type is established when a system is required to use the COScore in an one-way direction, whereas the second type connects the COScore with a CPS which inter-exchanges information in both directions.

Moreover, the connection may require the presence of a *gateway* if the system has a device which involves the use of a specific communication protocol different from the message exchange through the components’ input and output ports. If, on the contrary, the components can communicate with each other by using this port mechanism, the data is sent and received with the use of Web sockets through the communication web service of the COScore, as explained in Section 2. Next, the different connection alternatives are summarized:

- Low coupling connection from a CPS to the COScore. This type occurs when a component (or a system) is intended to send data to another component (belonging to the same or to other system) through the COScore. In the example scenario, the multimedia UI of the personal trainer sends the video data to the TV system of the monitored customer with this kind of connection.
- Low coupling connection from the COScore to a CPS. This connection is focused on solving the reception of data from another component (or a system) through the COScore. In the scenario, the UI showing the heart rate is connected in this manner.
- High coupling connection between a CPS and the COScore. This connection is used when a component of a CPS is required to establish a bidirectional communication with another component or it establishes a unidirectional communication with it but the COScore sends some feedback information. For example, the UI of the security system needs to (1) send the information about the alarm activation and (2) receive the trigger event if the window detects an intruder.
- Previous connection types but using a gateway as intermediary. As mentioned, a gateway is required if a component needs to communicate with a specific communication protocol. For example, the home automation sensor establishes a bidirectional communication with the UI of the same system through a gateway because the sensor is deployed on a KNX network [22].

In our approach, gateways are implemented as software components conforming the definitions of the background technology. Furthermore, this kind of component includes some communication methods which are not exposed by means of ports and therefore such operations are not represented in the models within provided or required interfaces. These methods are included as part of the code implementation with the aim of communicating with a physical device (or network) through a specific protocol. Nevertheless, these operations are usually invoked from the execution of the functionality related to the sending and receiving actions of the ports.

**Figure 5:**
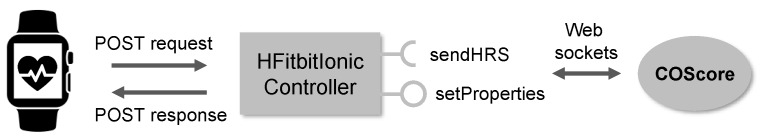
HFitbitIonicController component implementing the SW-Gateway.

The SW-Gateway of the scenario is a software component intended to communicate (a) with the heart rate sensor of the smart watch; and (b) with the UI that shows a graphical representation of the heart rate. The first interoperability process is performed through the gateway implementation and it includes POST messages between the smart watch and the gateway software component to obtain the value of the heart rate. The second interoperability process is accomplished by using the COScore services as intermediate through a communication port of a required interface (see the software component, implementing the SW-Gateway in Figure 5, named *HFitbitIonicController*).

The required interface of the *HFitbitIonicController* indicates that it must be connected to another component that provides the functionality for sending the value of the heart rate. In this case, the behavior of the gateway is not passive (waiting for a request), but it is pro-active for sending the value to other components periodically. To this end, the output communication port *sendHRS* of the required interface emits a message through a web socket (lines 1–10 of Listing 1). When the COScore receives the message, it is routed to the CPS with the UI which shows the heart rate. The function of this output port is activated when it receives a POST request from the smart watch (lines 12–21). This request can be answered because a proxy server is created to this end (lines 23–29).

Apart from gateways, the generic behavior to solve the interoperability between the devices of a solution formed by different CPSs is related to the controller interfaces of the components implementing the behavior of such devices. The *controller interface* contains the methods implementation of the functional interfaces of a component. Thus, parameter entries and return data from these methods are solved by input and output ports by using WebSockets [20] and the Socket.IO library [21]. When a *sender* component emits a message through an output port, it is received by the COScore. Then, the COScore checks the architectural representation of the solution, which describes the connection of all the components from the different CPSs. Next, the COScore emits a new message through a web socket to be received by the component connected with the *sender* (i.e., by the corresponding *receiver*).

Listing 1: Chunks of code of the SW-Gateway component implementation.


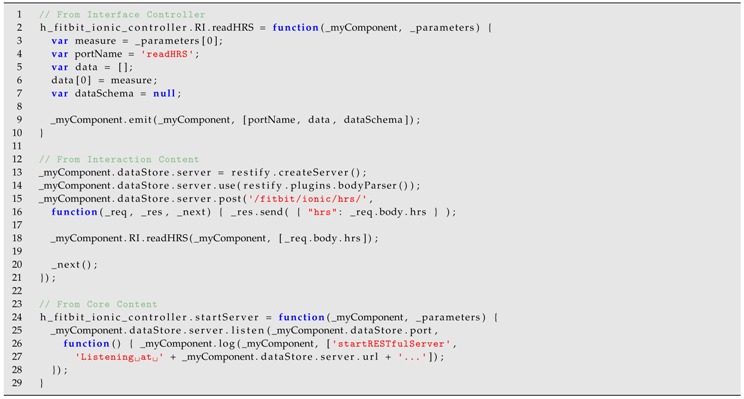


With this approach, a sender can be connected with multiple receivers and vice versa. Furthermore, input ports belonging to both types can be activated from the COScore with the need of waiting to the normal execution of the components’ business logic. As a consequence, component operations can be invoked as an API, considering and managing the indirect communications that may occur as a result of the activation of a certain operation.

Interoperability must be ensured even when the Internet connection is not available. For this reason, we have developed a local version of the COScore called ECOScore (from Embedded COScore). This version is not offered as a cloud service but is deployed in a single board computing (SBC) which must be installed in each cyber-physical system or in a common local network connecting different CPSs. As a consequence, the access to centralized cloud service will be filtered by the ECOScore: if the Internet is accessible, the requests are solved by the COScore and, in the other case, they are responded by the ECOScore.

## 4. Example Scenario

As previously described in the Introduction, the proposed scenario comprises various CPS systems. This scenario has been built through the utilization of a diversity of devices and technologies to illustrate the capacity of developed technology. Logically, as addressing comprehensive set-up in detail would be extensive, this example scenario focuses on certain elements of each system and of two possible interactions: (1) interaction between the devices belonging to two different cyber-systems in which human intervention does not figure and; (2) interaction between two humans with the support of several CPSs. The first kind of interaction is utilized to validate the application of our approach when two devices or subsystems communicate with each other. Both cyber-physical parts can use their own communication protocol but the software components (specifically, the controllers belonging to each technology) enable the interaction by using a common communication layer. The second kind of interaction allows us to validate how our approach is useful for enabling communication between users. In this sense, users are provided with software components, including interfaces with or without graphical representation, which establish communication channels between humans and smart home devices, and also between different humans acting as clients of a shared system.

### 4.1. Interaction 1: CPS–CPS Interaction

**Figure 6:**
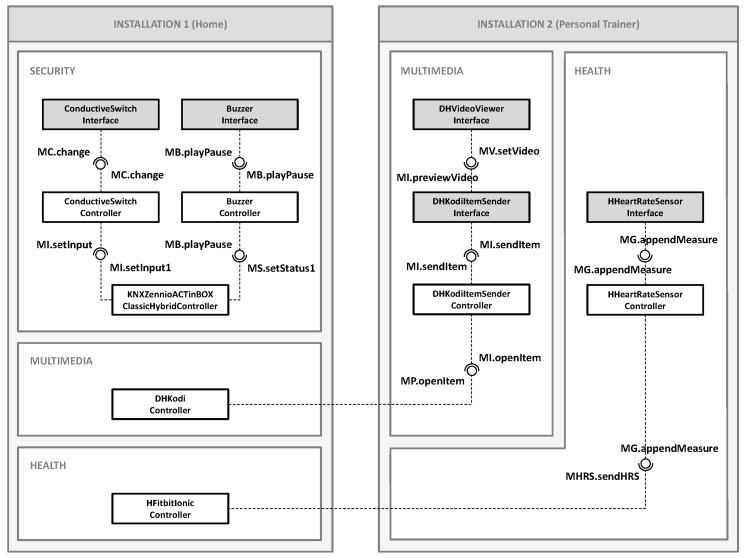
Partial representation of the example architecture.

The first interaction is related to the security system. Capacitive sensors are used on the exterior windows and doors of the house connected to Raspberry Pi boards. These sensors are connected through an electro-conductive paint that extends over the surface of the window or door, thus enabling potential difference to be measured and using the measurement to detect the presence of an individual; allowing it to act as an intrusion detector. In the event that this happens, the system should set off a sound alarm (a device belonging to a multimedia system), to signal the alert of an unwanted presence and act as a deterrent effect. Figure 6 partially represents the architecture of the COTSgets components as stated. This architecture includes only those components necessary for the two cases of interaction which are posed. Two different installations are interconnected: installation 1 corresponds to the home user, while installation 2 to the personal trainer. In both installations, it can be observed how the different components are grouped in the aforementioned systems.

Focusing attention on the security system of installation 1, it can be seen that a controller component has been incorporated into each of the physical devices (*ConductiveSwitchController*, *BuzzerController* and *KNXZennioACTinBOXClassicHybridController*) and, moreover, the corresponding interface components are linked (*ConductiveSwitchInterface* and *BuzzerInterface*). Also, in the figure, one can observe the different relationships between the components across their corresponding provided and required interfaces. Let us describe what happens since an intrusion is detected until an alarm sounds. In this situation, the following process takes place:

(1) Each time a modification in the potential difference is produced, the *ConductiveSwitchInterface* component transmits the data across the port of the required interface *ManageChange.change* to the connected port of the provided interface *ManageChange.change* of the *ConductiveSwitchController*.
(2) This, exceeding a true pre-established threshold and by means of the port of the required interface *ManageInput.setInput*, transmits the data through the port of the provided interface *ManageInput.setInput1* of the component *KNXZennioACTinBOXClassicHybridController* in order to activate the corresponding input.
(3) Next, the component *KNXZennioACTinBOXClassicHybridController* activates the output, thus communicating across the port of the required interface *ManageStatus.setStatus1* with the port of the provided interface *ManageBuzzer.playPause* of the *BuzzerController* component.
(4) Finally, the *BuzzerController* component communicates through the output port of the required interface *ManageBuzzer.playPause* with input port of the provided interface *ManageBuzzer.playPause* of the component *BuzzerInterface* signaling it to playback the alarm sound.

Of course, the alarm activation can be accompanied by other actions, like flashing the illumination lights of the affected room or recording camera images for a determinate period of time. Up to now, we have shown how devices of distinct technologies can interact, in this case, a capacitive sensor connected to a Raspberry Pi board with a KNX device and an alarm. Let’s see now how a human-human interaction occurs.

### 4.2. Interaction 2: Human–Human Interaction

**Figure 7:**
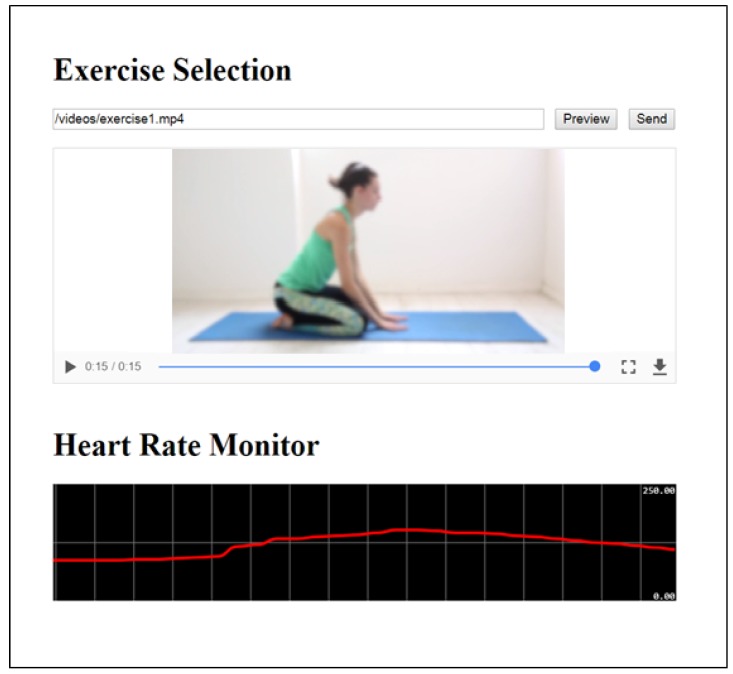
UI of the personal trainer.

The second interaction proposed is established between two users. Suppose that the home user has contracted the services of a personal trainer, whose interaction is carried out by remote access. During the activity the trainer monitors his/her client and, based on the data obtained, sends the next exercise to be done. To do the exercise correctly, the trainer will send a demonstration video. In this case, the multimedia systems come into play with healthcare. In installation 1, the home user has a television which is connected to a Raspberry Pi board, now configured as a multimedia center (specifically it has a version known as KODI installed with its API JSON-RPC activated). Moreover, the user has a smart watch (Fitbit Ionic model) which has a heart rate sensor to measure heart rate. The personal trainer can select and preview a video from among a series of videos from the installation 2 (a URL of an external video link can be introduced) in order to send it directly to the television of the customer through the multimedia center. Furthermore, the system permits the monitoring of heart rate in real time and thus prevents whatever risk. Figure 7 shows a screenshot of the trainer’s UI which comprises the components *DHKodiItemSenderInterface*, *DHVideoViewerInterface* and *HHeartRateSensorInterface*.

**Figure 8:**
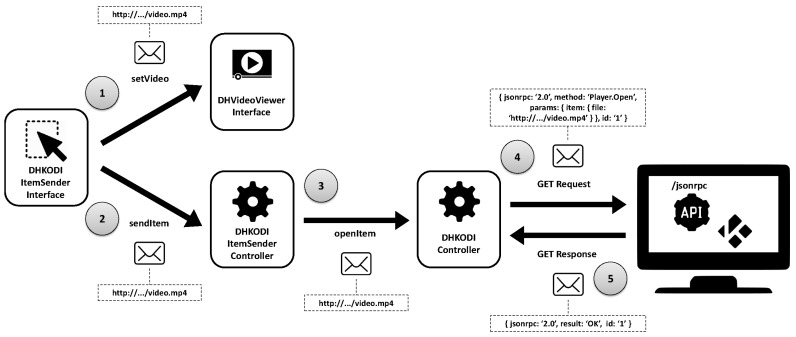
Communications of interaction human-to-human (first part).

Returning to the architecture of the components in Figure 6, *DHKodiController* component has been incorporated into the installation 1, which enables communication with the API JSON-RPC of KODI, and the previously mentioned *HFitbitIonicController*, which receives information and also permits its transmission to the watch. It is essential to highlight the infrastructure used to obtain the data from the watch sensors. On the one hand, it has been necessary to develop an application for the watch and the mobile phone (with the FitBit application installed) to which it is paired. On the other hand, the installation of a web server with a digital certificate has also been necessary (to provide secure connections) which acts as a proxy. The personal trainer’s installation (installation 2) is made up of the controller components *DHKodiItemSenderController* and *HHeartRateSensorController*, together with their interface components *DHKodiItemSenderInterface*, *DHVideoViewerInterface* and *HHeartRateSensorInterface*, which interact as shown in Figure 8:

(1) Optionally, the *DHKodiItemSenderInterface* component communicates through the port of the required interface *ManageItem.previewVideo* with the provided interface *ManageVideo.setVideo* of the component *DHVideoViewerInterface* if the trainer decides to preview a video (this last component will be charged with its reproduction).
(2) Once the decision to send the exercise video to the user is performed, the *DHKodiItemSenderInterface* interface component indicates the video to the *DHKodiItemSenderController* component, through the connection between the port of the required interface *ManageItem.sendItem* of the former and the port of the provided interface *ManageItem.sendItem* of the latter.
(3) Next, this last component sends the command to playback the video across the port of the required interface *ManageItem.openItem* to the port of the provided interface *ManagePlayer.openItem* of the *DHKodiController* component in the customer installation.
(4)–(5) To finalize, the *DHKodiController* component interacts with the API JSON-RPC of KODI (executing a GET operation and obtaining the response with the result) and the video starts on the user’s television.

**Figure 9:**
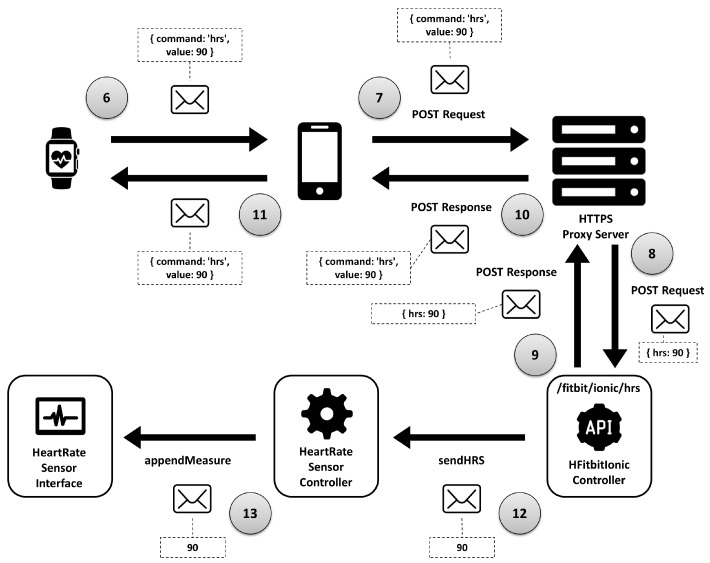
Communications of interaction human-to-human (second part).

The user views the video and, just before beginning the exercise, starts the application on the watch such that the trainer is able to monitor heart rate (Figure 9):

(6) The smart watch obtains the sensor data and sends it to the mobile.
(7) This device executes a POST request to the web server with the digital certificate, which includes the data read by the sensor.
(8) The server redirects the request to the REST web service offered by the *HFitbitIonicController* component.
(9)–(11) Now, this component sends a response (steps #9–#11) and communicates (through the port of the required interface *ManageHRS.sendHRS*) with the port of the provided interface *ManageGraph.appendMeasure* of the *HHeartRateSensorController*.
(12) It sends the value read by the sensor to the trainer’s UI.
(13) This last component, through the port of the required interface *ManageGraph.appendMeasure*, sends the new readings to the port of the provided interface *ManageGraph.appendMeasure* of the *HHeartRateSensorInterface* component.

Finally, this interface component will modify the graph visualized by the trainer. Through this example it is shown how the software components, as well as the interconnection of different devices and technologies within the same system, offer us the possibility of performing communication and interoperation processes between different systems.

## 5. Related Work

In the literature, there are different types of middleware solutions for the Internet of Things (event-based, service-oriented, agent-based, virtual-machine middlewares, tuple-space middlewares, database-oriented, etc.), although some approaches use a combination of these types to integrate heterogeneous devices [11]. In the case of event-based middlewares, it is usual to use the publish and suscribe pattern but, in some scenarios, it is better to use a message-oriented middleware thus relying on the messages and their formal structure [23,24]. An example of event-based middleware is RUNES [25], which is also component-based and intended to provide an architecture to get networked embedded systems. Another component-based architecture, but in this case service-oriented, is offered by the MUSIC middleware [26] that is focused on systems where dynamic changes occur in the context of service providers and consumers. The open source middleware provided by universAAL IoT [27] adds a semantic layer and uses three different communication channels (Context Bus, Service Bus and UI bus) to integrate heterogeneous devices, but it increases the complexity of using this solution and the development of new applications, in contrast to our approach. There are other middlewares that are specific for applications with the need of solving particular requirements instead of providing general purpose solutions. For example, the approach presented in [28] is focused on decreasing the transmission in the specific domain of smart-home applications. Our approach has taken into account this kind of middlewares and tries to improve them by proposing a component model based on Web technologies to support interoperability and extension features in dynamic and volatile systems with heterogeneous devices. The constraint of using a concrete component-based technology may define our solution as application-specific, but it has been developed with the aim of resolving the heterogeneity of different kinds of CPSs with the only restriction of using gateways and software components that must share the same communication technology based of web protocols.

The use of Internet-based middlewares can be oriented to resolve the interoperability between IoT devices that use different communication protocols. For example, we can use abstract representations of the systems and the communication protocols to create and deploy run-time solutions to IoT interoperability using only high-level models. For example, in [29] authors proposed a message language and automata models to enable the communication between different protocols. In a more recent research work [30], a translator architecture connecting service models is used to connect different devices that ‘speak’ different language within IoT applications based on Service Oriented Architectures (SOAs). In our case, model definitions are utilized as abstract representations and guidelines to implement components conforming the COScore background technology can manage. In addition, these models forces that communication and interoperability are accomplished by providing a common layer of interaction based on web sockets.

The concept of adaptive middlewares for cyber-physical systems highlights the need for a holistic view considering horizontal and vertical aspects that can be described through abstractions to solve the interoperability and integration of heterogeneous devices [31]. We also find the concept of emergent middleware to support interoperability [32]. This concept is different from the traditional middleware solutions, because it is based on a formal study of the networked systems involved in a scenario to (1) be able to calculate a mediator model that resolves the differences between the communication protocols and (2) automatically build the software that implements the mediator [33]. Related to this kind of model-driven middleware [34], the paper presented in [35] facilitates the creation of custom middlewares with characteristics that match the requirements of a specific domain through models which define the desired configuration. Furthermore, the use of models to abstract the representation of devices can be useful to develop and evaluate the adequacy of different alternatives in cyber-physical systems [36]. In our case, we have not created our solution as a middleware following a model-driven approach, but it is model-based since the components connected by our mediation software are defined by models, and the creation and execution of our systems are model-based processes. Thus, the main difference with other model-based solutions is the integration of MDE and CBSE paradigms to provide abstract representations of the smart home devices and the rest of software components which is used to create the communication layer based on web technologies.

The concept of the Web-of-Things (WoT) is based on IoT and tries to integrate the networked devices (i.e., things) into the Web by means of web technologies [37]. For example, RESTful principles can be used to integrate embedded devices from a cyber-physical systems [38]. In addition, heterogeneous sensor and actuator networks can be connected and discovered through an architecture based on RESTful web services [39]. Regarding model-based approaches, web and cloud computing technologies can be described from the abstract view of the WoT through model at run-time to create dynamic cyber-physical ecosystems [40]. Our approach is inspired by the abstraction concepts to deploy the software components which manage the CPSs using a web technology (in this case we have used Polymer web components) and they are managed by using RESTful web services. This web technology allows us to develop different types of components including those with user interface capabilities that enable the interaction with users. In contrast to WoT elements, the operations offered by our components are not only resource-oriented and they establish (through the definition of their provided or required interfaces) any type of action they can perform or that they need to be performed by another external component.

## 6. Conclusions and Future Work

Interoperability is a major problem related not only to those cyber-physical systems formed by heterogeneous IoT devices that use different communication protocols, but also to installations composed by a set of CPSs interconnected and sharing information. Some mechanisms concerning CBSE and MDE paradigms have proven their usefulness to develop this kind of system and ease the interoperability, and moreover, to maintain them and adapt their behavior to new requirements. Smart homes are a particular case of CPS and their application is growing in normal houses from the integration of existing subsystems that are working separately. This specific domain is an ideal scenario where different types of devices (with proprietary protocols) are interconnected and new elements are added to include new functionality. As a main part of these solutions, sensors are in charge of capturing the input information and sharing it with the rest of the elements of an installation. Furthermore, sensors are not only present as physical devices, but also are implemented as software components to (1) supplement the actions of existing physical elements; (2) emulate their actions if these devices are not installed; and (3) provide another sort of information that cannot be acquired by a physical sensor (e.g., temperature data obtained from a third-party web service).

In this paper we define a solution focused on enabling the interoperability between heterogeneous devices (physical and virtual) that form part of the same system and also between different CPSs. To this end, our approach proposes a set of design and implementation principles based on a component technology. Such technology includes the application of cloud service called COScore, which manages the software architectures that implement the cyber-physical systems. This management consists of the manipulation of the models representing the architectures and their realization to specific platforms. In this case, the technology also includes a platform-specific model to build and deploy the software architectures by using web components, in particular, implemented with Polymer. The solution for interoperability gathers (1) the unified communication through web sockets between all the software components that implement, control and show the behavior of the heterogeneous devices that form part of the CPSs; and (2) the use of gateways when a device or a system requires the utilization of a communication protocol different from the message exchange through the web sockets. Furthermore, we ensure the interoperability when there is no Internet connection by deploying a local version of the COScore called ECOScore. It is a reduced version of the cloud service which enables the communication between devices in the same network.

This approach is not valid for all cyber-physical systems and there are certain constraints (not too restrictive) to be able to apply our solution to any smart home installation. In the first place, the behavior of each device must be susceptible of being encapsulated in a black-box component which exposes their required and provided functionality by means of interfaces. Second, we need a repository containing the most common devices or, otherwise, developers must implement each new device as a Polymer component. Finally, for each specific network or communication protocol, we need to implement a gateway that allows us to interact with the connected devices.

As future work, we intend to develop and provide a set of tools supporting the design and development of interconnected CPSs that automatically generate (totally or partially) the implementation of the components according to our approach. This generation can be addressed by applying techniques of model-to-model (M2M) and model-to-text (M2T) transformations [41]. With the aim to obtain useful metrics that validate the efficiency of our approach, we will develop a set of experiments that evaluate the performance of different aspects of our approach, including response times, impact on software life-cycle, user experience in real smart home installations, or level of difficulty to develop different types of components, among other possible examples. In addition, to improve the present work, we will study the possible generation of open datasets from the interactions, events and information exchanged in this kind of system. This can be affordable if it is added as an implementation requirement of our components.
